# The Association between *RAD23B* Ala249Val Polymorphism and Cancer Susceptibility: Evidence from a Meta-Analysis

**DOI:** 10.1371/journal.pone.0091922

**Published:** 2014-03-18

**Authors:** Zhenjun Li, Yan Zhang, Xiaojiang Ying, Junmin Song, Ruoxin Zhang, Zhen Li, Hongliang Chen, Pingjiang Ye, Yi Shen, Weihuo Pan, Zhiliang Chen

**Affiliations:** 1 Department of Colorectal Surgery, Shaoxing People's Hospital, Shaoxing Hospital of Zhejiang University, Shaoxing, Zhejiang, China; 2 Department of Medical Oncology, Kunshan First People's Hospital Affiliated to Jiangsu University, Suzhou, Jiangsu, China; 3 Department of Colorectal Surgery, The First Affiliated Hospital of Zhengzhou University, Zhengzhou, Henan, China; 4 Department of Clinical Pharmacology, Barts and London School of Medicine and Dentistry, London, United Kingdom; Tulane University Health Sciences Center, United States of America

## Abstract

**Background:**

A number of studies have investigated associations of genetic variation in *RAD23B* Ala249Val (rs1805329 C>T) with cancer susceptibility; however, the findings are inconsistent. We performed a meta-analysis to acquire a more precise estimation of the relationship.

**Method:**

We searched literatures from PubMed, Embase and Web of Science. Pooled odds ratios (ORs) and 95% confidence intervals (CIs) were calculated to estimate the association between Ala249Val polymorphism and cancer risk.

**Results:**

A total of 23 studies consisting of 10837 cases and 13971 controls were included in this meta-analysis. Overall, no significant associations were found between *RAD23B* Ala249Val polymorphism and cancer risk (Val/Val vs. Ala/Ala: OR = 0.97, 95% CI = 0.75–1.25; Ala/Val vs. Ala/Ala: OR = 1.08, 95% CI = 0.96–1.22; recessive model: OR = 0.93, 95% CI = 0.76–1.14 and dominant model: OR = 1.07, 95% CI = 0.94–1.20). We did not find any significant associations in the further stratification analyses by cancer type, ethnicity and source of control.

**Conclusions:**

Despite some limitations, this meta-analysis indicates that it is unlikely that the *RAD23B* 249Val/Val polymorphism may contribute to the individual susceptibility to cancer risk. However, further advanced designed studies with larger sample size and different ethnicities should be conducted to confirm our results.

## Introduction

Cancer is one of the leading causes of death in economically developed countries as well as developing countries. The global burden of cancer continues to increase [1]. DNA damage is highly relevant to all aspects of oncology. Most mutations and large genomic alterations (deletions, translocations, loss of heterozygosity, and amplifications) that are relevant to cancer originate from DNA injury [2]. Four major DNA repair pathways exist in mammalian cells, in which nucleotide excision repair (NER) pathway is the most diverse and well-studied DNA repair system [3], [4]. The NER pathway plays important roles in the repair of bulky lesions, such as pyrimidine dimers, photo-products, larger chemical adducts and cross-links, as well as in the maintenance of genomic stability [4]. There are at least four steps of reaction (DNA damage recognition, incision of damaged DNA, repair of the gapped DNA, DNA ligation) and several key enzymes (XPC-RAD23B, CSB, XPA and XPF-ERCC1, etc.) involved in the NER pathway [4]–[6]. Polymorphisms in genes related to NER pathway may alter the DNA repair capacity and also play a role in carcinogenesis [7]. For example, the Lys939Gln and Ala499Val as well as the ploy (AT) deletion/insertion polymorphisms of the *XPC* gene were significantly associated with an increased overall cancer risk [8], [9]. The XPC binds to RAD23B, and forms the XPC-RAD23B complex, which plays important roles in the damaged DNA recognition and DNA repair initiation in the NER pathway [10].

*RAD23B* gene, also called *HR23B* or *HHR23B*, is located at chromosome 9q31.2. There are at least 1102 reported single nucleotide polymorphisms (SNPs) in the *RAD23B* gene region (http://www.ncbi.nlm.nih.gov/projects/SNP). *RAD23B* Ala249Val (rs1805329 C>T) is one of the important polymorphisms and it encoding protein involved in DNA damage recognition, which influence cancer susceptibility in individuals [11]. A number of studies have focused on the association between *RAD23B* Ala249Val polymorphism and caner risk [12]–[31], but the conclusions are still inconsistent. The possible reasons may be the small effect of the polymorphism on cancer risk and the relatively small sample size in each published study. Hence, we performed this meta-analysis combining the relevant published studies to draw a more precise estimation of the association.

## Materials and Methods

### Search strategy

We systematically searched PubMed, Embase and Web of Science using the search terms: “*RAD23B* or *HR23B* or *HHR23B*”, “polymorphism or variant or variation” and “cancer or carcinoma or tumor” (the last search was updated on November 2, 2013). All searched studies were retrieved, and their references were also checked for other relevant publications. Review articles and bibliographies of other relevant studies identified were searched manually to obtain additional eligible studies. Only the publications in English with full text available were included. If the same patient population was used in several publications, only the most recent study was included in this meta-analysis.

### Inclusion and exclusion criteria

The inclusion criteria were as follows: (a) evaluation of the association between *RAD23B* Ala249Val polymorphism and cancer risk, (b) case-control designed studies, (c) sufficient data provided for estimating odds ratios (ORs) and 95% confidence intervals (CIs).

The exclusion criteria were as follows: (1) study design, case-only or non-cancer subjects only studies; (2) duplicate of a previously published study; (3) deviation from Hardy-Weinberg equilibrium (HWE) in controls without further evidence of other polymorphisms of *RAD23B* gene or other genes.

### Data extraction

Two of the authors (Zhenjun Li and Yan Zhang) independently extracted the information from all the eligible publications according to the inclusion criteria. When the two authors had a disagreement on any elements such as the source of controls, ethnicity and etc., it was resolved by discussion between them. If consensus could not be reached, another author would be consulted to resolve the dispute and the majority of the votes made a final decision. The following data were collected from each study: first author's surname, publication year, country of origin, ethnicity, cancer type, source of controls, genotyping method, total number of cases and controls, and numbers of cases and controls with Ala/Ala, Ala/Val and Val/Val genotypes for the *RAD23B* Ala249Val polymorphism, respectively. Different ethnicities were categorized as Caucasian, Asian, Latino, African and more than one ethnicity. Source of controls were stratified to population-based and hospital-based. Cancer type was further categorized as respiratory tract, digestive tract, urinary system, breast, brain and other cancer. We did not define any minimum number of patients to include in our meta-analysis.

### Statistical methods

We used the goodness-of-fit chi-square test to evaluate HWE for each study, and *P*<0.05 was considered as deviation from HWE. Crude ORs with their corresponding 95% CIs were used to assess the strength of association between the *RAD23B* Ala249Val polymorphism and cancer risk. The pooled ORs were performed in homozygous model (Val/Val vs. Ala/Ala), heterozygous model (Ala/Val vs. Ala/Ala), recessive model (Val/Val vs. Ala/Ala + Ala/Val), and dominant model (Ala/Val + Val/Val vs. Ala/Ala), respectively. Chi-square-based Q-test was used to check heterogeneity assumption. *P* values greater than 0.1 for the Q-test suggest a lack of heterogeneity among studies, hence we used the fixed-effects model (the Mantel-Haenszel method) to calculate the pooled OR estimate of the each study [32]. Otherwise, the random-effects model (the DerSimonian and Laird method) was used [33]. We performed subgroup analyses by variables of cancer type, ethnicity and source of controls. Sensitivity analysis was performed to assess the accuracy and stability of the results. Potential publication bias was estimated by the funnel plot, in which the standard error of log (OR) of each study was plotted against its log (OR). An asymmetric plot suggests a possible publication bias. Funnel plot asymmetry was assessed by the method of Egger's linear regression test, a linear regression approach to measure funnel plot asymmetry on the natural logarithm scale of the OR. The significance of the intercept was determined by the t-test suggested by Egger (*P*<0.05 was considered as statistically significant) [34]. All the statistical tests were performed with STATA version 11.0 (Stata Corporation, College Station, TX). All *P* values were two-sided, and *P*<0.05 was considered statistically significant.

## Results

### Study characteristics

**Figure 1:**
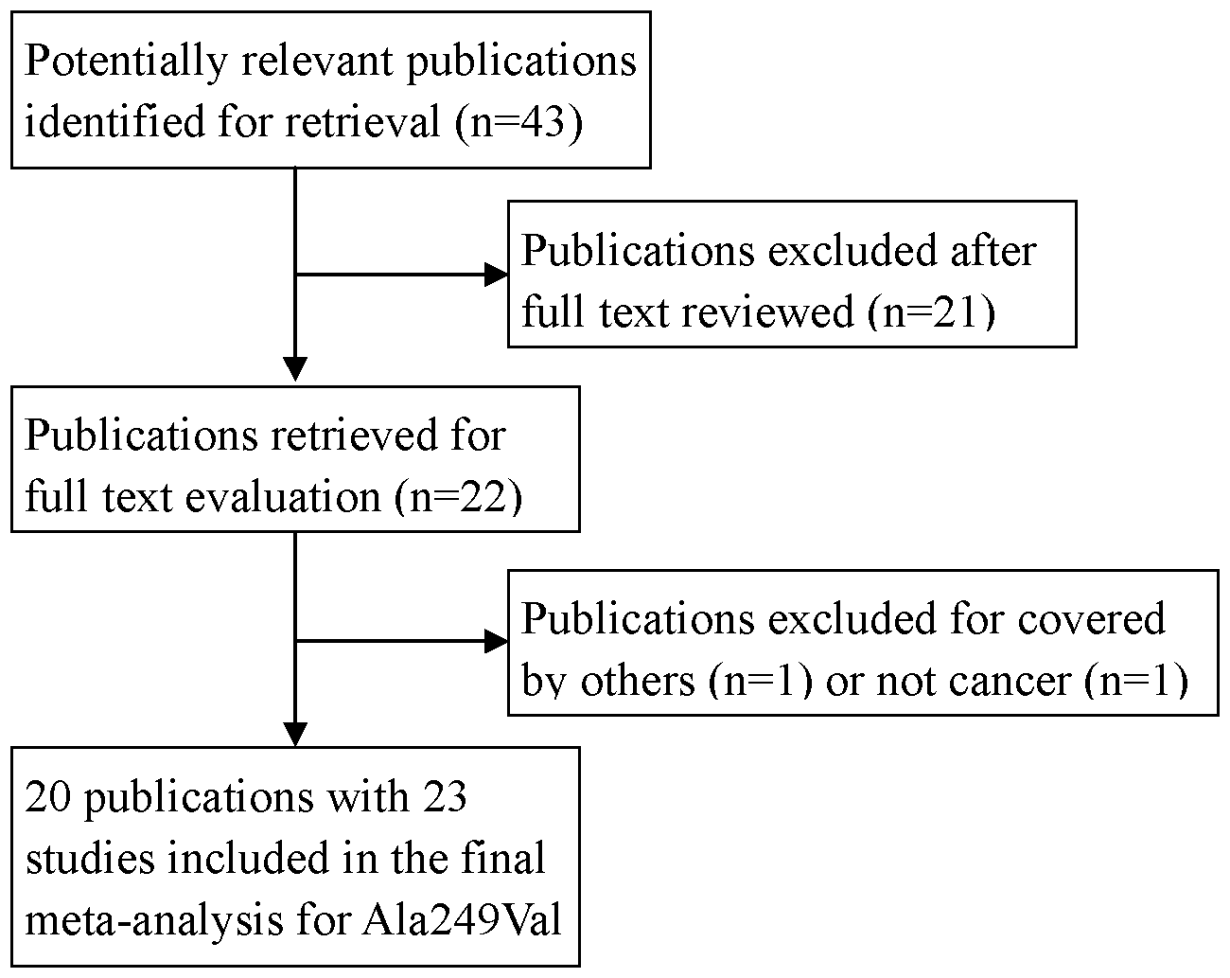
Flow chart for the process of selecting the 23 included studies for this meta-analysis.

We identified 43 potentially relevant publications after a comprehensive literature screening from the previous mentioned databases. After assessing the publications according to the inclusion/exclusion criteria, 20 of the 43 publications met the inclusion/exclusion criteria and were included in the final analysis (**Figure 1**) [12]–[31]. One publication was excluded for it focused on oral premalignant lesions, which cannot be classified as cancer [35]. Another one [36] was excluded for overlapping data with a previous study [19]. The remaining 21 publications were not association studies. As a result, a total of 20 publications including 10837 cases and 13971 controls were used in the meta-analysis. For publications reporting results for more than one ethnic group [15] or cancer type [27], the results were analyzed and reported separately. Therefore, 20 publications were reported as 23 studies. **Table 1** summarizes the studies identified and their main characteristics. The sample sizes ranged from 61 to 1267 for cases, and from 111 to 2405 for controls, in which four studies focused on the type of respiratory tract cancer, digestive tract cancer, urinary tract cancer, brain tumor as well as other cancers, and three on breast cancer. There were 17 studies on Caucasians, three studies of Asians, two studies of Latinos, one study of Africans, and one with more than one ethnicity, respectively. Cases were confirmed by histologically for 12 studies, and controls were matched for sex and age for 13 studies. Of the 23 studies included in our final analysis, 11 were population-based, 12 were hospital-based. The distribution of genotypes in the controls did not deviate from HWE, except for three studies [17], [23], [24]. In light of the distribution of genotypes for other DNA repair genes in the controls was consistent with HWE, so we included these three studies in our final analysis to enlarge our sample size and minimize the selection bias.

**Table 1:** Characteristics of studies included in the meta-analysis.

Surname	Year	Country	Ethnicity	Cancer type	Cancer type	Cases/controls	Control source	Genotype method	MAF	HWE
Shen [12]	2005	China	Asian	Lung	respiratory tract	118/111	PB	Real-time PCR	0.20	0.702
Huang [13]	2006	USA	More than one ethnicity	Colorectal	digestive tract	685/704	PB	Real-time PCR	0.20	0.308
Landi [14]	2006	European	Caucasian	Lung	respiratory tract	279/296	HB	APEX	0.25	0.219
Mechanic [15]	2006	USA	African	Breast	Breast	763/679	PB	TaqMan	0.06	0.413
Mechanic [15]	2006	USA	Caucasian	Breast	Breast	1267/1133	PB	TaqMan	0.18	0.520
Shen [16]	2006	USA	Caucasian	NHL	others	477/557	PB	TaqMan	0.18	0.093
Millikan [17]	2006	USA	Caucasian	Melanoma	others	1200/2405	PB	TaqMan	0.20	0.004
García-Closas [18]	2006	Spain	Caucasian	Bladder	urinary system	1137/1127	HB	Real-time PCR	0.15	0.656
Wu [19]	2006	USA	Caucasian	Bladder	urinary system	607/595	PB	TaqMan	0.19	0.271
Chang [20]	2008	USA	Latino	Lung	respiratory tract	113/299	PB	Illumina	0.27	0.569
Lin [21]	2008	USA	Caucasian	Renal cell	urinary system	322/335	PB	TaqMan	0.20	0.838
Zhang [22]	2008	China	Asian	Biliary Tract	digestive tract	410/779	PB	TaqMan	0.18	0.620
Abbasi [23]	2009	Germany	Caucasian	Larynx	respiratory tract	248/647	PB	Real-time PCR	0.20	0.030
McKean-Cowdin [24]	2009	USA	Caucasian	Glioblastoma	Brain	986/1925	HB	MassARRAY	0.19	0.028
Pan [25]	2009	USA	Caucasian	Esophageal	digestive tract	382/452	HB	TaqMan	0.18	0.069
Michiels [26]	2009	France	Caucasian	Bladder	urinary system	189/316	HB	Illumina	0.18	0.467
Rajaraman [27]	2010	USA	Caucasian	Glioma	Brain	323/444	HB	TaqMan	0.83	0.130
Rajaraman [27]	2010	USA	Caucasian	Meningioma	Brain	117/444	HB	TaqMan	0.74	0.130
Rajaraman [27]	2010	USA	Caucasian	Neuroma	Brain	61/444	HB	TaqMan	0.57	0.130
Ibarrola-Villava [28]	2011	Spain	Caucasian	Melanoma	others	599/379	HB	MassARRAY	0.15	0.320
Tomoda [29]	2012	Japan	Asian	Liver	digestive tract	763/679	HB	MassARRAY	0.26	0.085
Perez-Mayoral [30]	2013	USA	Latino	Breast	Breast	183/373	HB	TaqMan	0.22	0.133
Santos [31]	2013	Portugal	Caucasian	Thyroid	others	106/212	HB	Taqman	0.16	0.485

MAF, Minor allele frequency; HWE, Hardy-Weinberg equilibrium; PB, Population based; HB, Hospital based; APEX, Arrayed primer extension; NHL, non-Hodgkin lymphoma.

### Meta-analysis result

**Figure 2:**
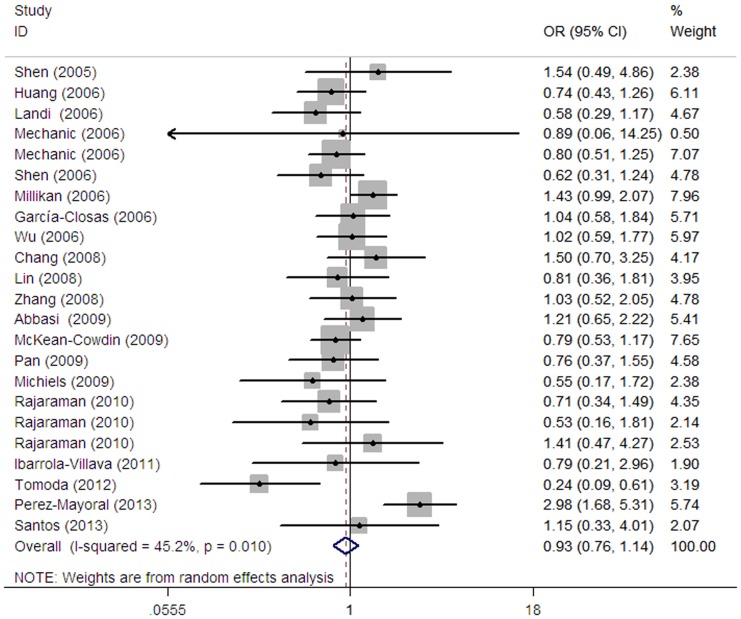
Forest plot for overall cancer risk associated with the RAD23B Ala249Val polymorphism by a recessive model for each of the 23 published studies. For each study, the estimates of ORs and their corresponding 95% CIs were plotted with a box and a horizontal line. The symbol filled diamond indicates pooled OR and its corresponding 95% CI.

The main results of this meta-analysis are shown in **Table 2** and **Figure 2**. Overall, no significant associations were found between *RAD23B* Ala249Val polymorphism and cancer risk when all studies were pooled into the meta-analysis (Val/Val vs. Ala/Ala: OR = 0.97, 95% CI = 0.75–1.25; Ala/Val vs. Ala/Ala: OR = 1.08, 95% CI = 0.96–1.22; recessive model: OR = 0.93, 95% CI = 0.76–1.14 and dominant model: OR = 1.07, 95% CI = 0.94–1.20). No significant associations were observed when stratified by cancer type, ethnicity and source of controls.

**Table 2:** Meta-analysis of the association between *RAD23B* Ala249Val polymorphism and cancer risk.

Variables	No. of studies	Homozygous	*P*_het_ a	*I* ^2^	Heterozygous	*P*_het_ a	*I^2^*	Recessive	*P*_het_ a	*I* ^2^	Dominant	*P*_het_ a	*I* ^2^
		VV vs. AA			AV vs. AA			VV vs. AA+AV			AV+VV vs. AA		
All	23	0.97 (0.75–1.25)	<0.001	64.6	1.08 (0.96–1.22)	<0.001	72.9	0.93 (0.76–1.14)	0.010	45.2	1.07 (0.94–1.20)	<0.001	77.0
Cancer type													
Respiratory tract	4	1.11 (0.65–1.89)	0.138	45.6	1.10 (0.81–1.50)	0.073	57.0	1.07 (0.68–1.69)	0.238	29.0	1.11 (0.80–1.55)	0.031	66.1
Digestive tract	4	0.67 (0.38–1.18)	0.053	60.9	1.09 (0.90–1.33)	0.127	47.4	0.66 (0.40–1.10)	0.096	52.8	1.02 (0.80–1.30)	0.028	66.9
Breast	3	1.89 (0.34–10.45)	<0.001	92.5	1.51 (0.64–3.61)	<0.001	95.8	1.43 (0.46–4.43)	0.002	84.1	1.54 (0.61–3.86)	<0.001	96.5
Urinary system	4	0.97 (0.69–1.36)	0.748	0.0	1.14 (1.00–1.29)	0.966	0.0	0.93 (0.66–1.31)	0.761	0.0	1.12 (0.99–1.27)	0.905	0.0
Brain	4	0.78 (0.56–1.08)	0.579	0.0	0.93 (0.81–1.07)	0.554	0.0	0.79 (0.58–1.09)	0.670	0.0	0.91 (0.80–1.04)	0.449	0.0
Others	4	1.00 (0.62–1.61)	0.199	35.6	0.90 (0.80–1.01)	0.985	0.0	1.03 (0.64–1.66)	0.193	36.6	0.92 (0.82–1.03)	0.889	0.0
Ethnicity													
Caucasian	16	0.92 (0.79–1.08)	0.533	0.0	1.03 (0.95–1.11)	0.198	22.6	0.92 (0.78–1.07)	0.562	0.0	1.01 (0.94–1.09)	0.177	24.5
Asian	3	0.76 (0.23–2.56)	0.006	80.6	1.15 (0.74–1.77)	0.026	72.7	0.72 (0.25–2.06)	0.018	75.2	1.10 (0.65–1.86)	0.003	83.2
Latino	2	3.13 (0.73–13.42)	0.004	87.9	2.05 (0.45–9.38)	<0.001	95.9	2.23 (1.15–4.33)	0.163	48.7	2.20 (0.48–10.04)	<0.001	96.3
African	1	0.86 (0.05–13.77)	/	/	0.69 (0.48–0.98)	/	/	0.89 (0.06–14.25)	/	/	0.69 (0.48–0.98)	/	/
More than one ethnicity	1	0.75 (0.44–1.29)	/	/	1.06 (0.84–1.33)	/	/	0.74 (0.43–1.26)	/	/	1.02 (0.82–1.27)	/	/
Control source													
PB	11	1.04 (0.87–1.25)	0.496	0.0	1.04 (0.93–1.17)	0.044	46.6	1.03 (0.86–1.23)	0.459	0.0	1.04 (0.94–1.15)	0.062	43.1
HB	12	0.88 (0.53–1.45)	<0.001	78.9	1.12 (0.90–1.40)	<0.001	82.2	0.83 (0.57–1.21)	0.002	62.5	1.09 (0.86–1.38)	<0.001	85.9

PB, Population based; HB, Hospital based.

a *P* value of the Q-test for heterogeneity test.

### Sensitivity analysis

We deleted one single study involved in the meta-analysis at a time to reflect the impact of the individual data-set to the pooled ORs, and the corresponding pooled ORs were not materially altered (data not shown), indicating that our results were statistically robust.

### Publication bias

We used Begg's funnel plot and Egger's test to assess the publication bias of the literatures. The shape of the funnel plot revealed some evidence of obvious asymmetry and we found one study may lead to publication bias [30]. Therefore the Egger's test was used to provide further statistical evidence of funnel plot symmetry. We did not find any evidence of publication bias from the results after this study was excluded (*P = *0.426 for Val/Val vs. Ala/Ala; *P* = 0.071 for Ala/Val vs. Ala/Ala; *P = *0.458 for recessive model; and *P = *0.087 for dominant model, respectively).

## Discussion

We performed this meta-analysis including 10837 cases and 13971 controls to estimate the association between *RAD23B* Ala249Val polymorphism and cancer risk. We did not find any significant association between this polymorphism and the overall cancer risk. Although the exact mechanisms of how *RAD23B* Ala249Val polymorphism affects cancer risk at the molecular level remain unknown, the established knowledge on the structural and biological functions of the *RAD23B* gene may imply the potential effect of this polymorphism.

*RAD23B* gene is located at chromosome 9q31.2. The protein encoded by *RAD23B* is one of the two human homologs of *Saccharomyces cerevisiae Rad23*. RAD23B and XPC proteins bind to form an XPC-RAD23B heterodimeric subcomplex [6], [37], which plays a key role in DNA damage recognition in the NER global genome repair pathway. It has been demonstrated that polymorphisms of DNA repair genes may lead to DNA repair capacity alteration, sequentially contribute to cancer susceptibility [38]. Hence, variants in both genes may interact to hinder NER and increase the risk of cancer [7].

In the subgroup analysis based on ethnicities and source of controls, we did not find any statistical significance. The potential reason for the association discovered in study may not be reproduced across all ethnic groups may be ascribed to the ethnicity difference which may expose to different environment as well as dietary difference. Some of the findings may be due to chance because studies with small sample size may have insufficient statistical power to detect a slight effect [39]. Considering the limited studies and population numbers of each ethnicity included in the meta-analysis, our results should be interpreted with caution.

We also found two recent meta-analyses focused on the overall cancer risk and *XPC* gene polymorphisms [8], [9]. The XPC can bind with RAD23B to form the XPC-RAD23B complex which plays an important role in damaged DNA recognition [10]. He et al. [8] found that the *XPC* Lys939Gln polymorphism was significantly associated with overall cancer risk from 62 studies with a total of 25708 cancer cases and 30432 controls, they also found the Ala499Val polymorphism was associated with overall cancer risk with evidence from 34 studies including 14877 cases and 17888 controls. Likewise, Dai et al. [9] also found the *XPC* PAT +/+ carriers may have an increased overall cancer risk from 32 publications with 10214 cases and 11302 controls. However, in this study, we did not find any evidence for the association between *RAD23B* Ala249Val polymorphism and overall cancer risk, which may be due to this polymorphism exposing a weak or nearly no effect on cancer risk. Carcinogenesis is a complex process, and different cancer types may have different mechanisms. In addition, it is possible that the sample size for any given cancer is not sufficient to detect any association.

Cigarette smoking is one of the risk factors for lung cancer as well as other cancers such as bladder cancer [40], [41]. Tobacco smoke contains at least 55 carcinogens that can generate reactive oxygen species and sequentially lead to mutations [42]. The carcinogens can interact with human DNA and cause DNA damages, and if left unrepaired, such DNA damages can induce mutations and initiate tumorigenesis [43]. Yin et al. [44] found that heavy cigarettes smokers may have an increased lung cancer risk. Exposure to certain environmental factors might be required for detecting the association of DNA repair polymorphisms and cancer risk.

Several limitations of this meta-analysis should be addressed. First, the numbers for each type of cancer and sample sizes were relatively small, with possible insufficient statistical power to investigate the real association; second, our results were based on unadjusted estimates, whereas a more precise analysis should be conducted if raw data from each individual study were available. This would allow for the adjustment by other co-variants including age, gender, smoking status, drinking status, BMI, virus infection, environmental factors, and other lifestyle. Moreover, we only included the articles written in English, which may potential miss some articles.

In conclusion, this meta-analysis indicated that *RAD23B* Ala249 Val polymorphism was not associated with cancer susceptibility. However, interactions of gene variants and environmental risk factors, such as smoking, drinking, infection, and sun exposure should also be considered in the analysis. Such studies including more samples with different ethnicities, environmental factors, and sufficient biological evidence for the SNP functions may lead to a better, comprehensive understanding of the association between the *RAD23B* Ala249Val polymorphism and cancer risk.

## Supporting Information

Checklist S1 **PRISMA Checklist.**
(DOC)
